# Lineage analysis of human papillomavirus type 39 in cervical samples of Iranian women

**DOI:** 10.1186/s12985-021-01619-8

**Published:** 2021-07-22

**Authors:** Neda Hosseini, Zabihollah Shoja, Arash Arashkia, Amir-Hossein Khodadadi, Somayeh Jalilvand

**Affiliations:** 1 Department of Virology, School of Public Health, Tehran University of Medical Sciences, Tehran, 14155 Iran; 2 Department of Virology, Pasteur Institute of Iran, Tehran, Iran

**Keywords:** Human papillomavirus, Type 39, Lineage, Cervical cancer

## Abstract

**Background:**

The data with regards to the regional variants of distinct HPV types is of great value. Accordance with this, this study aimed to investigate the sequence variations of E6 gene and long control region of HPV 39 among normal, premalignant and malignant cervical samples in order to characterize the frequent HPV 39 variants circulating in Tehran, Iran.

**Methods:**

In total, 70 cervical samples (45 normal, 16 premalignant, and 9 malignant samples) infected with HPV 39 were analyzed by nested-PCR and sequencing.

**Results:**

Our results revealed that all samples belonged to A lineage. Almost all sequences (98.6%) were classified in A1 sublineage and only one sample (1.4%) was A2 sub lineage.

**Conclusions:**

Our findings showed that lineages A, sublineage A1, is dominant in Tehran, Iran. However, the small sample size was the most important limitations of this study. Further studies with larger sample size from different geographical regions of Iran are necessary to estimate the pathogenicity risk of HPV 39 variants in this population.

**Supplementary Information:**

The online version contains supplementary material available at 10.1186/s12985-021-01619-8.

## Introduction

Cervical cancer is reported as the fourth most frequent cancer among women worldwide [1]. Human papillomavirus (HPV) is considered as the etiological agent of cervical cancer. Although more than 40 HPV types can infect the anogenital tract, 14 HPV types, designated as the high-risk HPV types including 16, 18, 31, 33, 35, 39, 45, 51, 52, 56, 58, 59, 68 and 73, associate with progression to cervical cancer [2–6]. Among high-risk HPV types, HPV 16 and 18 are leading cause and responsible for almost 72% of cervical cancer [7]. Most studies on the prevalence of HPV types has indicated that HPV 16 and 18 are the most prevalent types in women with normal cervical cytology and invasive cervical cancer in Iran [8–13]. A meta-analysis was revealed that the five most common HPV types are HPV 16, 18, 31, 39, and 45 in Iranian women with cervical cancer [14]. Indeed, HPV 16, 18, 31, 39, and 45 are account for 52%, 14.1%, 5%, 3.3%. and 3.3% of cervical cancer, respectively [14].

A distinct HPV type is considered when the DNA sequence of the L1 gene was differ from that of any other characterized type by at least 10%. Isolates of the same HPV type are designated to as lineage and sublineage when the nucleotide sequences of the L1 gene differ by less than 1–10% and 0.5–1%, respectively. Up to date, HPV 39 has two distinct lineages A and B; which lineage A include 2 different sublineages A1 and A2 [15].

While the co-evolution of HPV 16 and HPV 18 variants and human populations is well characterized [4, 16–19], the geographic associations for variants of other types remains to be inconclusive. To better understand of an association between the distinct variants of HPV 39 and ethnicity further studies are needed in the world.

The data with regards to the regional variants of distinct HPV types is of great value as it would provide a rational for future studies on their evolution, epidemiology, pathogenicity, and biology. Whereas the spreading of HPV types is identified in Iran, there is much less known about HPV variants. Previous studies were investigated the common HPV 16, 18, 31, and 45 variants in Iran [19–21]. Regard to above-mentioned data that HPV 39 is one of the five most common HPV types in Iranian women with cervical cancer [14], this study aimed to investigate the frequent HPV 39 variants circulating in Iran. According to this fact that E6 gene can only separate A and B lineages from each other, long control region (LCR) were analyzed to distinguish two distinctive sublineages A1 and A2.

## Material and methods

### Study population

To characterize lineages and sublineages of HPV 39 a cross-sectional study was designed from 2018 to 2020. One-hundred and fifty-six formalin-fixed paraffin-embedded (FFPE) samples (98 invasive cervical cancer samples and 58 premalignant cervical lesions) were obtained from Immam-Khomeini hospital in Tehran. Moreover, 55 ThinPrep Pap Test specimens (10 premalignant and 45 normal samples) that were HPV 39 positive were collected from three referral laboratories in Tehran from 2018 to 2020. These samples were previously genotyped by Cobas assay or INNO-LiPA® HPV Genotyping assay. All of study subjects sign the informed consent and all information regard to their medical records were stored on secure system.

### Variant analysis of HPV 39 based on E6 gene and long control region (LCR)

DNA extraction from ThinPrep Pap Test specimens was carried out by the High Pure Viral Nucleic Acid Kit (Roche Diagnostics GmbH, Roche Applied Science, Mannheim, Germany) according to the manufacturer’s instruction. Genomic DNA from formalin-fixed paraffin-embedded (FFPE) samples was isolated using phenol–chloroform assay according to previously published procedure [22].

Detection of HPV genome was investigated in 156 FFPE samples using nested-PCR with MY09/MY11 and GP+5/GP+6 primer pairs, respectively, to amplify a 150 bp fragment of L1 gene. To genotype, all HPV positive samples were subjected to sequencing by BigDye® Terminator v3.1 Cycle Sequencing Kit and a 3130 Genetic Analyzer Automated Sequencer as specified by Applied Biosystems manuals (Foster City, CA). Nucleotide sequences were edited using Bioedit software and converted to FASTA format. Finally, edited sequences were blasted using the Blast server (http://www.ncbi.nlm.nih.gov/blast/) to find HPV genotypes. All of HPV positive samples that were genotyped by sequencing also screened by nested-PCR with sequence-specific primers of HPV 39 to amplify of E6 gene (Table 1).

**Table 1:** The list of primers were designed by oligo 7 software and used in this study

Target gene	Name of primer	Sequence of primer (5′–3′)	Nucleotide position	Amplicon size (bp)	References
E6	39-E6-F1	AGTAACCGAAAACGGTCAGGA	39–59	641	
	39-E6-R1	TCGTGACATACAAGGTCAACCG	659–680		This study
	39-E6-F2	CAGGACCGAAATCGGTGGAT	55–74	616	
	39-E6-R2	ACAAGGTCAACCGGCTGTAT	652–671		
LCR	39-LCR-F1	ACTATAGGTCCCCGAAAGCG	7077–7096	752	
	39-LCR-R1	AGTATAGGTATGTATGCCCAACC	7807–7829		This study
	39-LCR-F2	ACTTCCTCGTCCTCAGCTACTA	7110–7131	704	
	39-LCR-R2	GCCCAACCTATTTCGGTTGCAT	77,930–7814		

The complete sequence of HPV 39 E6 gene (nucleotide [nt] 107–583) was investigated using nested-PCR with the primer pairs were shown in Table 1. The PCR reaction was achieved in a 50 μl reaction mixture including 100–200 ng of DNA template, 2 mM MgCl_2_, 50 μM of each dNTP, 10 pmol of each primer, and 2 U of Taq DNA polymerase. PCR amplification cycles were as follow for both round: 35 cycles of 95 °C for 20 s, 55 °C for 40 s and 72 °C for 50 s. A reaction mixture without template DNA, as a negative control, was included in every set of PCR run (Additional file 1: Figure S1).

The LCR region of HPV 39 genome was amplified by nested-PCR with primer pairs 39-LCR-F1 and 39-LCR-R1 for first round and 39-LCR-F2 and 39-LCR-R2 for the second round (Table 1). The PCR reaction was carried out in a 50 μl reaction mixture including 100–200 ng of DNA template, 50 μM of each dNTP, 1.5 mM MgCl_2_, 10 pmol of each primer, and 2 U of Taq DNA polymerase. PCR amplification cycles were as follow for both round: 35 cycles of 95 °C for 20 s, 56 °C for 50 s and 72 °C for 50 s. A reaction mixture without template DNA, as a negative control, was included in every set of PCR run (Additional file 2: Figure S2).

To analyze of HPV 39 variants, the PCR products were addressed to bidirectional direct sequencing as described above. All sequences obtained in this study are available at http://www.ncbi. nlm.nih.gov/ with accession numbers MW390245-MW390314 and MW390315-MW390384 for E6 gene and LCR, respectively. To identify lineages and sublieages of the studied samples, sequences were aligned to reference sequences with accession number M62849, KC470239, and KC470247 designated for A1, A2, and B, respectively [15]. Phylogenetic tree was constructed using the maximum likelihood method by Mega software version 6 [23]. The reliability was measured by calculation of bootstrap with 1000 replicates. The reference sequences of A1, A2, and B [15] were also retrieved from GenBank database.

### Statistical analysis

The statistical analysis was performed by Mantel–Haenszel test (Epi Info 7, Statistical Analysis System Software) and the p-value less than 0.05 was considered statistically significant.

## Results

HPV 39 was detected in 15 FFPE samples (6 premalignant and 9 malignant samples). In total, 70 HPV 39-positive cervical samples of normal (n = 45), premalignant (n = 16), and malignant (n = 9) specimens were investigated in this study. The mean age of studied groups was 51.5, 37.5, and 30 years old in malignant, premalignant, and normal samples, respectively.

**Fig. 1:**
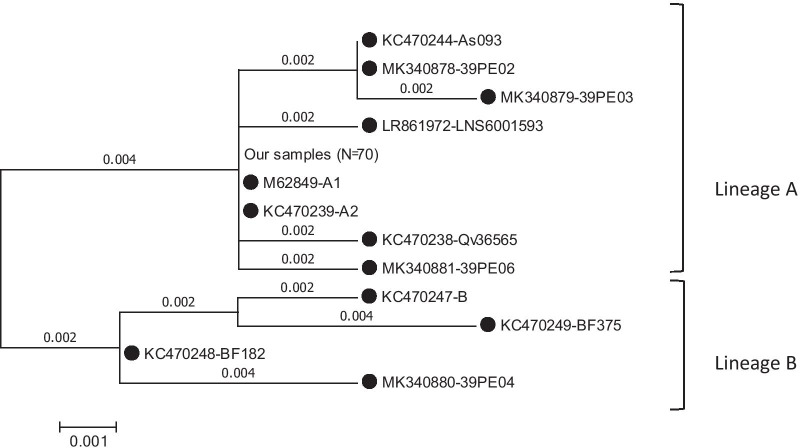
Phylogenetic tree analysis of HPV E6 gene was conducted in MEGA6 by using the Maximum Likelihood method based on the Kimura 2-parameter model [23]. The accession number of reference sequences used in this study were as follows: M62849; KC470232; KC470238; KC470239; KC470244; KC470246; KC470247; KC470248; KC470249; MK340878; MK340879; MK340880; MK340881; and LR861972 that were indicated by black circle. The bootstrap value was considered to be 1000 replicates

The complete E6 gene (nt 107-583) of 70 HPV 39-positive samples were sequenced and compared with the HPV 39 E6 prototype sequence (GeneBank accession number M62849). As indicated in Table 2, nucleotide substitutions at positions of G78T, G151T, C260G, T305C, and A453G are specific for lineage B. Phylogenetic tree analysis of HPV E6 gene was conducted in MEGA6 by using the Maximum Likelihood method based on the Kimura 2-parameter model [23]. Phylogenetic tree analysis of 70 samples were indicated that no nucleotide substitutions were happened in the E6 gene and all samples were belonged to lineage A (Fig. 1). Consequently, no amino acid changes were occurred in our samples (Table 2).

**Table 2:**
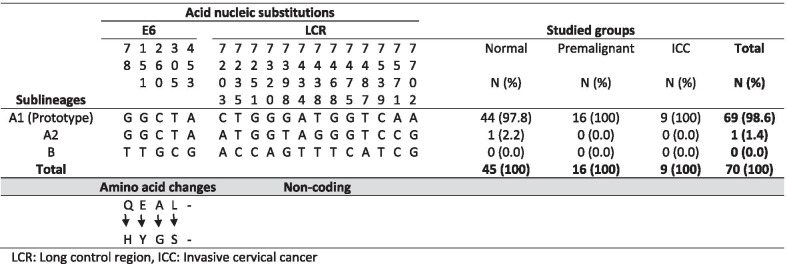
The frequency of HPV 39 sublineages identified in normal, premalignant, and invasive cervical cancer samples of Iranian women, regard to nucleotide substitutions at E6 gene and Long control region (LCR) as well as amino acid changes in E6 gene

**Fig. 2:**
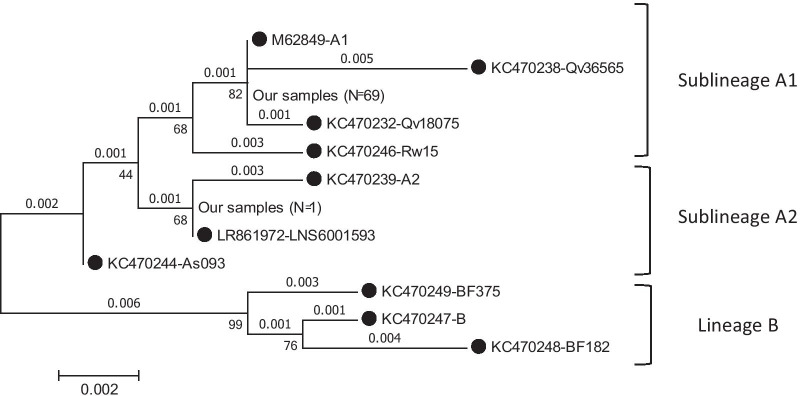
Phylogenetic tree analysis of HPV long control region was conducted in MEGA6 by using the Maximum Likelihood method based on the Kimura 2-parameter model [23]. The accession number of reference sequences used in this study were as follows: M62849; KC470232; KC470238; KC470239; KC470244; KC470246; KC470247; KC470248; KC470249; and LR861972 that were indicated by black circle. The bootstrap value was considered to be 1000 replicates

To distinguish sublineages A1 and A2 from each other, the partial sequence of LCR was investigated. Regard to LCR analysis, five nucleotide substitutions were found in only one of our studied samples at positions of C7203A, G7398T, T7438G, A7571C, and A7702G. Phylogenetic tree analysis of HPV LCR was conducted in MEGA6 by using the Maximum Likelihood method based on the Kimura 2-parameter model [23]. Phylogenetic tree analysis was revealed that almost all sequences (98.6%) were classified in sublineage A1 and only one sample (1.4%) was belonged to sublineage A2 (Fig. 2, Table 2).

As shown in Table 1 and Fig. 2, all of premalignant and malignant cervical samples were classified in sublineage A1. In normal group, 44 out of 45 samples (97.8%) were belonged to sublineage A1 and one samples (2.2%) was classified in sublineage A2. However, no statistically significant differences were found in this regard among three studied groups (*P* > 0.05).

As indicated in Table 3, 50 out of 70 samples (71.4%) were found to be infected with at least two different HPV types. In most of multiple HPV-infected samples (64%), another high-risk HPV types were detected and in 36% of samples a low-risk HPV type was found. Regard to histological type, multiple HPV infections were detected in 77.8%, 50%, and 77.8% of malignant, premalignant, and normal cases, respectively. Interestingly, infection with at least a high-risk HPV type in studied groups were found to be 85.7%, 50%, and 62.8% in malignant, premalignant, and normal samples, respectively (Table 3).

**Table 3:** The prevalence of multiple infection in HPV 39-positive samples of Iranian women with normal, premalignant, and invasive cervical cancer

Total	Normal	Premalignant	Malignant	
	Studied groups			
20 (28.6)	10 (20.5)	8 (50.0)	2 (21.2)	Single infection
50 (71.4)	35 (77.8)	8 (50.0)	7 (77.8)	Multiple infection
**32 (64.0)**	**22 (62.8)**	**4 (50.0)**	**6 (85.7)**	High-risk (HR) types
7 (14.0)	2 (5.8)	1 (12.5)	4 (57.1)	HPV 16
7 (14.0)	5 (14.2)	1 (12.5)	1 (14.3)	HPV 18
18 (36.0)	15 (42.8)	2 (25.0)	1 (14.3)	Non 16/18 HR types
**18 (36.0)**	**13 (37.2)**	**4 (50.0)**	**1 (14.3)**	Low-risk types

The total of high-risk and low-risk HPV types are indicated by bold

## Discussion

It is well recognized that the distribution of HPV 16 and 18 variants can be different geographically due to the evolution associated to the population ethnicity [16, 24–27]. However, the geographic relations for variants of other types remain inconclusive. In this regard, this study investigated the sequence variations of E6 gene and LCR of HPV 39 in order to find the frequent HPV 39 variants in normal samples as well as premalignant and malignant lesions of the cervix from Iranian women.

Almost uniform distribution of HPV 39 variants was found in normal and premalignant/malignant groups, as lineage A was dominant. Our result is consistent with few studies that conducted in some geographical regions of the world. One study in the United States of America was investigated the HPV 39 lineages in 479 samples as the most of samples belonged to lineage A (99%) and B lineage was only detected in 1% [28]. Another study shown that although lineage A was dominant in Costa Rica (67% A1 and 33% A2 sublineages), only lineage B was found in Burkina Faso [29]. In women from Shanghai, indicated that all three sublineages of HPV 39 are present as follow: sublineage A1 was dominant (81.2%) and the remaining of cases were belonged to sublineages A2 and B in 12.5% and 6.3%, respectively [30].

It is suggested that like HPV 16-related alpha-9 species variants, isolates of alpha-7 species have shown co-divergence of archaic Hominid and HPV variants. It seems that rapid expansion of the host population causing viral variant lineages and sublineages that had occurred almost 0.2–1.0 million years ago [29]. Indeed, the distinction coevolution of HPV 16 variants with closely related ancestral human populations together with introgression of certain archaic alleles related to innate immunity and keratinocytes differentiation into the human genomes might let to the dominancy of HPV 16 A lineage in modern human ancestor population [31].

Viral variants of each high-risk HPV types show differences in infectivity, long-term persistence, development of precancer lesions, and invasion of HPV containing cell [15]. Indeed, it is indicated that distinct HPV 16 lineages have different risks for development of cervical cancer. Several studies found that HPV 16 variants of non-European lineages (B, C, D) have stronger oncogenic potential than the European lineage A [18, 32–34]. In this study, however, no statistically significant differences were found by distinct lineages or sublineages of HPV 39 in three studied groups. Concordance with our result, finding of one study was revealed that no difference was observed in the increased risk of CIN2/3 regard to HPV 39 sublineages [28]. Moreover, regard to this point that multiple infection with another high-risk HPV types, particularly HPV 16 and 18 was common in malignant samples; it seems that probably HPV 16 or 18 played an important role in development of cervical cancer in Iranian women rather than HPV 39.

An association between population-based oncogenicity of HPV 39 variants and host genetic variations, particularly HLA class I and II alleles, could be assumed. Although in this regard no data was found for HPV 39, it is indicated that the E-G350 variant of HPV 16 had 4–fivefold higher risk for cancer development among Swedish women with HLA-B*44, HLA-B*51, or HLA-B*57 alleles [25]. A significant link between different E6 variants of HPV 16 and HLA class II alleles was also found for three distinct alleles (DRB1*1501, DRB1*1502 and DQB1*0602) in Japanese women, as DRB1*1501 and DQB1*0602 alleles were significantly higher among patients with the prototype variant while DRB1*1502 was increased in patients with D25E variants compared with controls [35].

The most important limitations of this study were found to be the small sample size and multiple infections.

## Conclusion

Our findings showed that lineages A, sublineage A1, is dominant in Tehran, Iran. HPV variant studies have been made important data that could help better understanding the carcinogenicity of different HPV types at molecular level. However, the small sample size and multiple infections were the most important limitations of this study. Further studies with larger sample size of single infection with HPV 39 from different geographical regions of Iran are necessary to estimate the pathogenicity risk of HPV 39 variants in this population. The characterization of HLA in Iranian women with cervical cancer is highly recommended as it may shed a new light to understand a link between HPVs variants and genetic background.

## Supplementary Information

**Additional file 1. Figure S1:** Gel electrophoresis to detect a 616 bp of E6 gene of HPV39. M: 100 bp+3K marker and N: negative control.

**Additional file 2. Figure S2:** Gel electrophoresis to detect a 704 bp of long control region of HPV39. M: 100 bp+3K marker and N: negative control.

## Data Availability

Data available within the article.

## Abbreviations

HPV: Human papillomavirus
LCR: Long control region
FFPE: Formalin-fixed paraffin-embedded
