# Supplementary figures and images for: Lineage analysis of human papillomavirus type 39 in cervical samples of Iranian women
Source: Virol J. 2021 Jul 22;18:152. doi: 10.1186/s12985-021-01619-8 (PMC8296747; doi:10.1186/s12985-021-01619-8)

## Slide 1
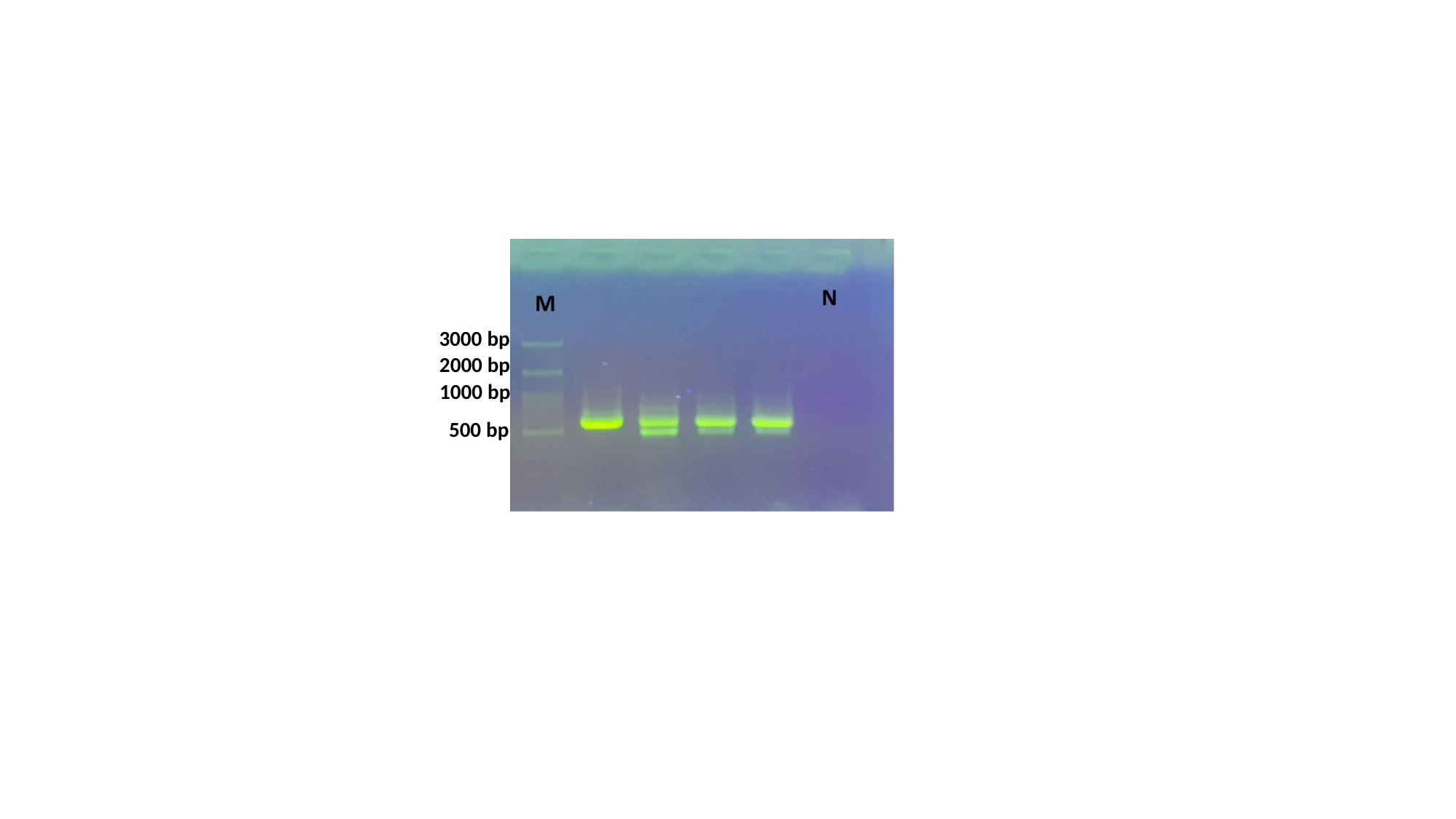

Supplement: Supplementary file 1 — Additional file 1. Figure S1: Gel electrophoresis to detect a 616 bp of E6 gene of HPV39. M: 100 bp+3K marker and N: negative control. [file 12985_2021_1619_MOESM1_ESM.pptx]

## Slide 1
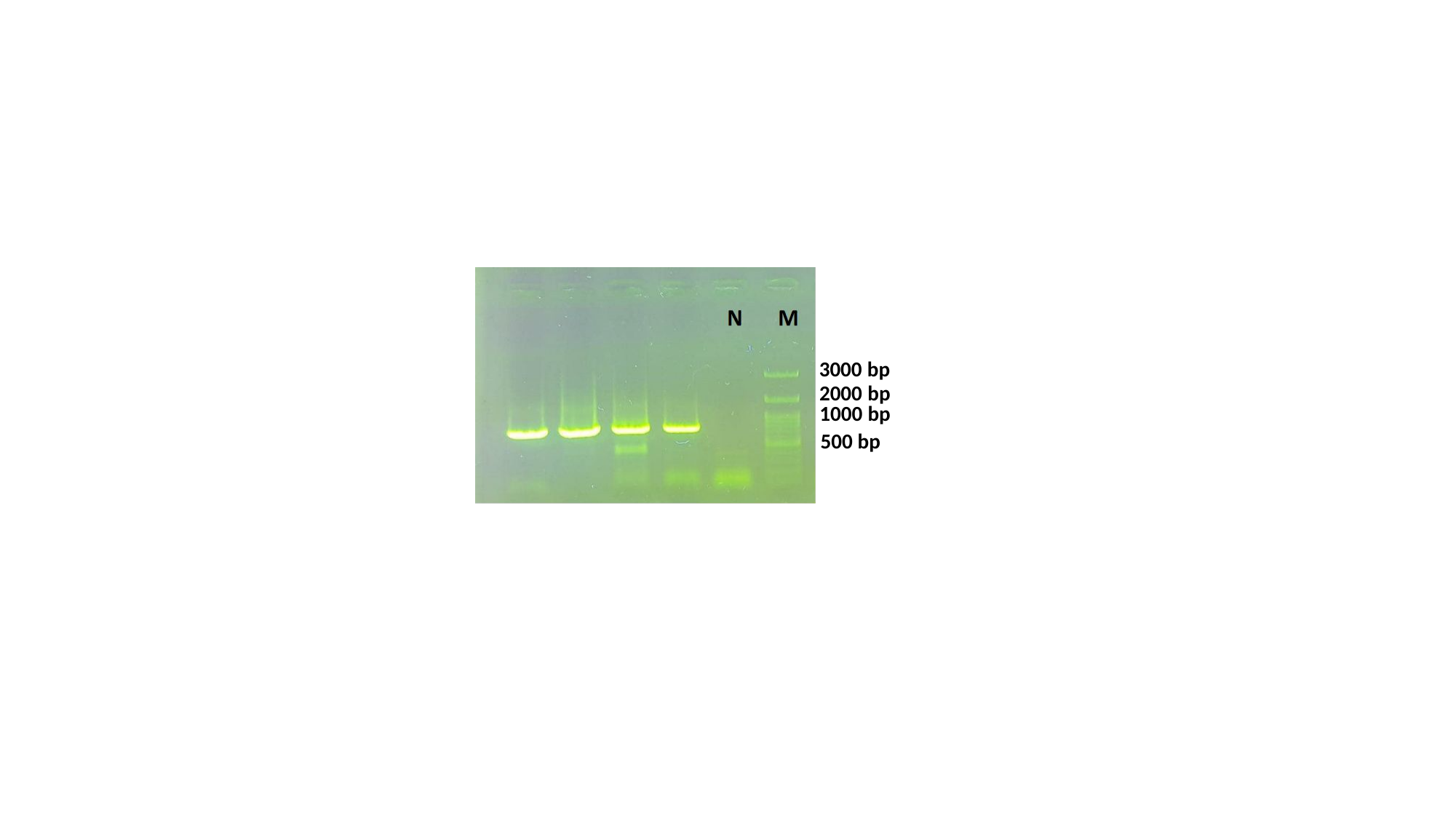

Supplement: Supplementary file 2 — Additional file 2. Figure S2: Gel electrophoresis to detect a 704 bp of long control region of HPV39. M: 100 bp+3K marker and N: negative control. [file 12985_2021_1619_MOESM2_ESM.pptx]
